# Development of *in-Situ* Al-Si/CuAl_2_ Metal Matrix Composites: Microstructure, Hardness, and Wear Behavior

**DOI:** 10.3390/ma9060442

**Published:** 2016-06-02

**Authors:** Mahmoud M. Tash, Essam R. I. Mahmoud

**Affiliations:** 1 Department of Mechanical Engineering, University of Prince Sattam bin Abdulaziz, AlKharj 11942, Saudi Arabia; 2 Mechanical Engineering, King Khalid University, Abha 61413, Saudi Arabia; emahoud@kku.edu.sa; 3 Welding and NDT Laboratory, Manufacturing Technology Department, Central Metallurgical Research and Development Institute (CMRDI), 87 Helwan, Cairo 11421, Egypt; 4 Mining, Petroleum and Metallurgical Engineering Department, Cairo University, Giza 12613, Egypt

**Keywords:** metal-matrix composites (MMCs), Al-Si alloys, copper powder, *in-situ* intermetallics, heat treatments, casting

## Abstract

In the present work, *in-situ* metal matrix composites were fabricated through squeeze casting. The copper particles were dispersed with different weight percentages (3%, 6%, 10%, and 15%) into Al-12% Si piston alloy. Also, heat treatments were performed at 380 °C and 450 °C for holding times of 6 and 18 h. The microstructures, X-ray diffractometer (XRD) pattern, hardness, and wear characteristics were evaluated. The results showed that these copper particles have reacted with the aluminum under all of the aforementioned processing conditions resulting in the formation of fine copper aluminide intermetallics. Most of the intermetallics were CuAl_2_, while AlCu appeared in a small ratio. Additionally, these intermetallics were homogenously distributed within the alloy matrix with up to 6% Cu addition. The amounts of those intermetallics increased after performing heat treatment. Most of these intermetallics were CuAl_2_ at 380 °C, while the Cu-rich intermetallics appeared at 450 °C. Increasing the holding time to 18 h, however, led to grain coarsening and resulted in the formation of some cracks. The hardness of the resulting composite materials was improved. The hardness value reached to about 170 HV after heat treating at 380 °C for 8 h. The wear resistance of the resulting composite materials was remarkably improved, especially at lower additions of Cu and at the lower heat treatment temperature.

## 1. Introduction

Aluminum-silicon alloys and their metal matrix composites are widely used in aerospace and automobile industries due to their low density, good mechanical properties, and corrosion resistance [1]. These materials have found applications in the manufacturing of various automotive engine components, such as cylinder blocks, pistons, and piston insert rings where adhesive wear (or dry sliding wear) is a predominant process [2]. The pistons for high-speed engines are primarily made of aluminum alloys which contain about 11%–13% silicon and ~1% for each Cu, Ni, and Mg [3]. A piston is the key part of an engine as it works under high temperature, high pressure, and corrosive and wearing conditions while running with high speed [4]. To reduce the hydrocarbon (HC) emission, the piston top must be very thin [5]. In order to satisfy all these severe conditions, piston materials must have high strength, high toughness, and be light weight. Several strengthening technologies, such as pressurization, have been developed in recent years to strengthen piston alloys. However, it should be pointed out that the results of these techniques could not satisfy all the recent application requirements [4]. Obviously, new techniques are required to increase the strength of the Al piston alloys. For this purpose, adding hard particles to the Al alloy (forming metal matrix composites (MMCs)) and decreasing grain size are considered optimum solutions. 

Al-based MMCs, reinforced with ceramics or metallic particles were developed as an alternative to materials with superior strength–weight and strength–cost ratios, high stiffness, and excellent thermal stability, which have great effects on improving wear, creep, and fatigue resistance. However, the poor toughness and the extra cost of metal matrix composites relative to aluminum alloys impose serious restrictions on their applications, especially at high volume fractions of the reinforcement [6]. Increasing the reinforcement volume fraction of the MMCs can significantly improve the strength and stiffness of the composites on one hand, but it drastically decreases the toughness and ductility on the other hand [7]. This can be attributed to an increase in the severity of the triaxiality of stress in the matrix, which results in an earlier onset of void nucleation in the matrix and at the particle/matrix interface [7]. On the other hand, it is widely recognized that the mechanical properties of metal matrix composites (MMCs) are controlled by the type, size, and volume fraction of the reinforcement phase(s), and the nature of the matrix-reinforcement interface. Superior mechanical properties can be achieved when fine, thermally stable, and hard reinforcement(s) with good and clean interfacial bonding are dispersed uniformly in the metal matrix. As suggested by several authors [8], such characteristics can be obtained when the reinforcements are formed inside the matrix during MMCs fabrication *(i.e.*, *in-situ* intermetallics) [9].

*In-situ* intermetallic compounds provide a new family of reinforcement phases (CuAl_2_, TiAl_3_, FeAl_3_, *etc.*) which possesses high specific strength, high specific modulus, and excellent wear properties at both ambient and elevated temperatures. These reinforcements are formed in the matrix by reaction of the added element(s) with each other or with the matrix, so the resulting dispersed particles can be expected to be thermally stable and have strong interfacial bonding with the matrix [10]. Copper is one of the ideal elements to be added to aluminum to form *in-situ* MMCs, due to its low cost and the high wear resistance of the Al-Cu intermetallics [11]. It was reported by. Dubourg *et al.* [11] that the Al-Cu intermetallics layer was fabricated by pre-placing the copper powder on the aluminum surface where it was then heated beyond the melting point of the substrate by a high energy laser beam. The resulting surface layer showed high hardness and good wear resistance [11]. 

A variety of methods for producing MMCs on the industrial scale have been developed, including powder metallurgy (PM) [12], high-energy milling [13], and severe plastic deformation [14], which are considered to be a forms of solid state processing. The major critical problems facing these processes are the contamination of results from powder preparation and the complexity of fabrication steps. Moreover, machining is required to obtain the desired final shape. The other group of fabrication processes for MMCs is liquid-state processing, which includes infiltration techniques, stirring techniques, rapid solidification, as well as some *in-situ* fabrication methods such as the liquid-gas bubbling technique [15]. These processes offer some advantages compared with solid-state processing, such as their energy-efficiency and cost-effectiveness [16]. However, non-homogeneous reinforcement distribution or particles agglomeration in the molten matrix or during solidification, and pore formation are considered to be critical problems facing the fabrication of MMCs by liquid-state processes [17]. One of the liquid state processes that can be utilized in producing MMCs is squeeze casting. In this process, the applied pressure and the instantaneous contact of molten metal with the die surface resulted in rapid heat transfer that yields a porous free casting with mechanical properties approaching the wrought product [18].

The present work aimed to obtain a new Al-alloy-based *in-situ*-composite material by squeeze casting that possessed excellent mechanical properties for piston application.

## 2. Experimental Procedures

In the present study, Al-12% Si Piston alloy (1.03% Cu, 0.9% Mg, and 1.55% Ni) was used as a matrix material. Pure copper powder with an average particle size of 5 µm was added to the matrix with different weight percentages of 3%, 6%, 10%, and 15%. The alloy was melted at 710 ± 5 °C. The added particles were heated to 100 °C for 10 min before dispersion into the alloy matrix in order to improve the interfacial bonding between the matrix and dispersed particles, and to facilitate the distribution of the dispersed particles inside the alloy matrix. After mixing, the melt was poured into a steel mold, and fixed pressure was applied. After cooling, heat treatment was performed for samples at temperatures of 380 and 450 °C for different holding times (6 and 18 h) followed by air cooling. Table 1 summaries the experimental conditions employed in the present study. 

The microstructure study was carried out using an optical microscope (Olympus optical microscope with digital camera, Tokyo, Japan) and scanning electron microscope (SEM) (JEOL JSM-5410 (SEM) JED-2140 (EDX) at 20KV, JEOL USA Inc., Peabody, MA, USA). Also, the samples were analyzed with an X-ray diffractometer (XRD) (D8 Discover with GADDS system, Bruker Corporation, Karlsruhe, Germany) to identify the phases that were originally found or *in situ* formed during casting and heat treatment processing. The hardness of the product was also measured with Vickers hardness tester (DVK-2 Matsuzawa Vicker hardness, Matsuzawa Co., Ltd., Akita, Japan). The wear behavior of the samples was evaluated using a pin-on-disk dry sliding wear tester (Pin on Disc Wear Testing Machine (TR-20, DUCOM), Indiamart Co., Noida, India). A stationary sample with a diameter of 2.5 mm was held against a rotating disk with a rotational speed of 265 rpm for 15 min. The tests were carried out with a fixed load of 2 kg applied to the pin. The specimens were weighed before and after the test with a sensitive electronic balance. The differences in weight before and after the wear test were measured and recorded. The untreated base metal was selected as the reference material for the wear test. For hardness and wear tests, each value represents the average of five readings. 

## 3. Results and Discussion

### 3.1. Effect of Addition of Copper Powder

**Figure 1:**
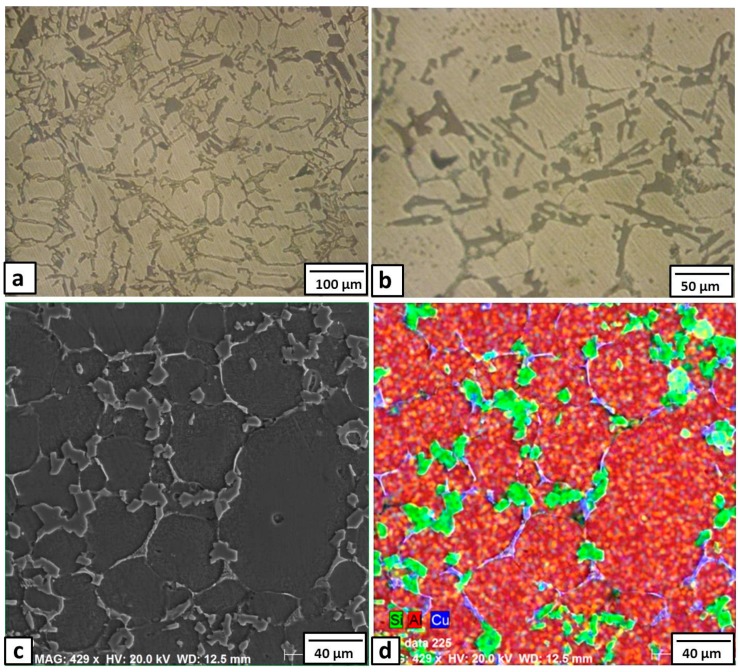
Optical micrographs of the Al-12% Si; (**a**) base metal; (**b**) base metal +3% Cu; (**c**) enlarged scanning electron microscope (SEM) image of base metal +3% Cu; and (**d**) EDS element analysis of image (**c**).

The basic microstructure of the Al-12% Si base metal, as shown in Figure 1a, consists of a eutectic structure of short silicon constituent that appeared at the grain boundaries of the α-phase Al matrix. This dendrite microstructure can be mainly attributed to the high silicon content of the matrix (11.56 wt %), approaching the eutectic composition (12.6%). The microscopic appearance of the microstructure of Sample 1 (with 3% Cu added) is shown in Figure 1b.

**Figure 2:**
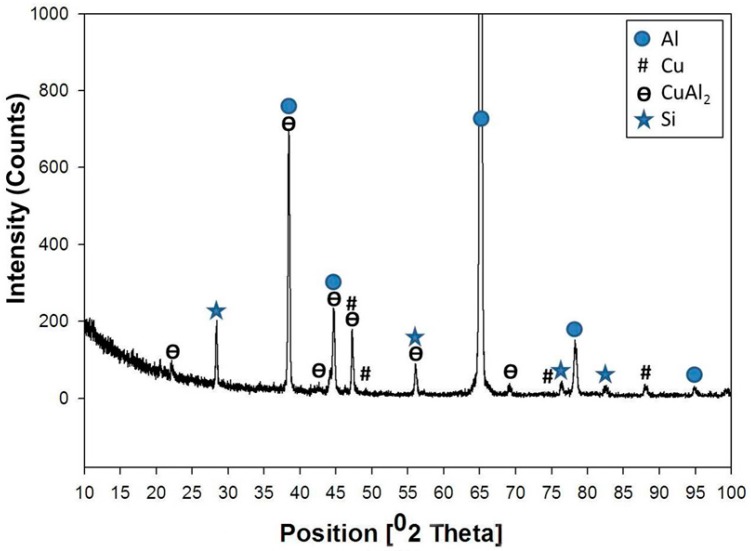
X-ray diffractometer (XRD) patterns obtained from of the formed composite after addition of 3% Cu.

Generally, in comparison with the base metal microstructure (Figure 1a), the micrographs obtained in Figure 1b show a new fine interdendritic structure distributed at the α-phase grain boundaries in addition to the normal short silicon dendrites. To clarify these new fine interdendrites, an SEM image and EDS element mapping is shown in Figure 1c,d, respectively. The mapping of the SEM micrograph shows that the Si constituents (green color) were modified from the short needles to a more rounded and morphology. At the same time, the added copper (blue color) also appeared in the grain boundaries of the Al α- grains. Moreover, the atomic concentrations of the Cu and Al estimated from the EDS spectra at the blue phase were about 31% and 65%, respectively. These concentrations suggest the formation of CuAl_2_ intermetallics. The typical XRD pattern for this sample (3% Cu) is shown in Figure 2. The XRD analysis revealed the presence of Al, and Al-Si eutectic, as the major peaks. In addition, small diffraction peaks of CuAl_2_ intermetallic phase were detected, which proved that the Cu particles had reacted with the Al matrix forming CuAl_2_ intermetallics, as suggested by the EDS analysis.

**Figure 3:**
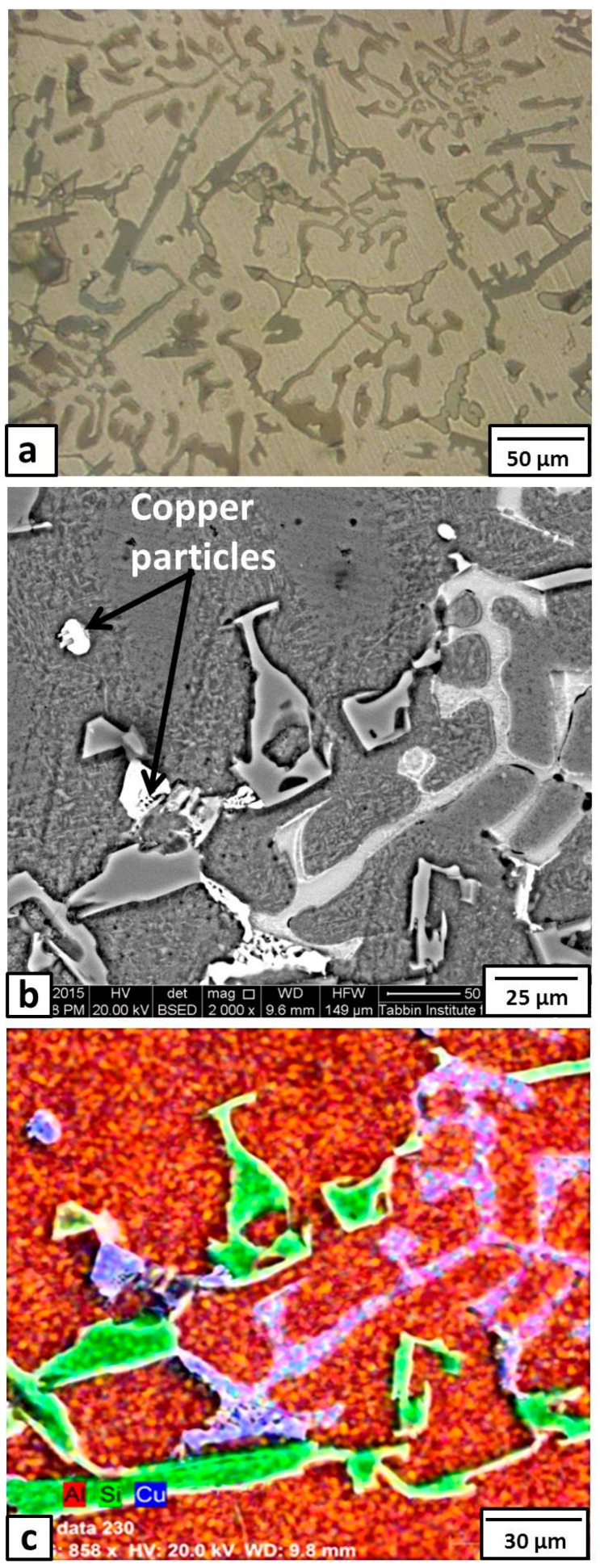
Microstructure of the formed composite after addition of 6% Cu; (**a**) optical micrographs; (**b**) enlarged SEM image; and (**c**) EDS element analysis of image (**b**).

The microstructure of Sample 2 (with 6% Cu added) is shown in Figure 3. There is no great difference in the optical microstructure (Figure 3a) with Sample 1 (Figure 1a). Many intermetallics appeared on the Al grain boundaries. The observed particles that were distributed in the matrix were almost round in shape. These were probably copper particles. The enlarged SEM image (Figure 3b), with EDS element mapping (Figure 3c), of this condition (6% Cu) showed many Si (green color) and Cu (blue color) dendrites. Moreover, the EDS atomic concentrations of the Cu at the locations of the aforementioned round particles (shown by arrows in Figure 3b) and the blue eutectic phase were about 93% and 68%, respectively, suggesting that the round particles were copper and the blue eutectic phase was CuAl_2_ intermetallic compound.

**Figure 4:**
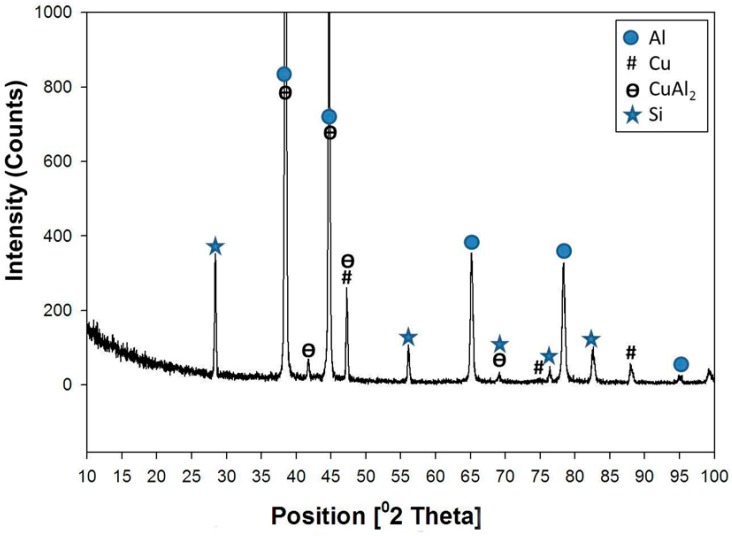
XRD patterns obtained from of the formed composite after addition of 6% Cu.

The XRD pattern obtained from Sample 2 (6% Cu added) is shown in Figure 4. The structure consisted of mixtures of Al, Al-Si eutectic, CuAl_2_, AlCu, and Cu with different ratios. The large intensities of the Al and Al-Si eutectic peaks were mainly due to the matrix. The appearance of the Cu peaks observed in XRD patterns indicates that there is some non-reacted Cu in the matrix.

**Figure 5:**
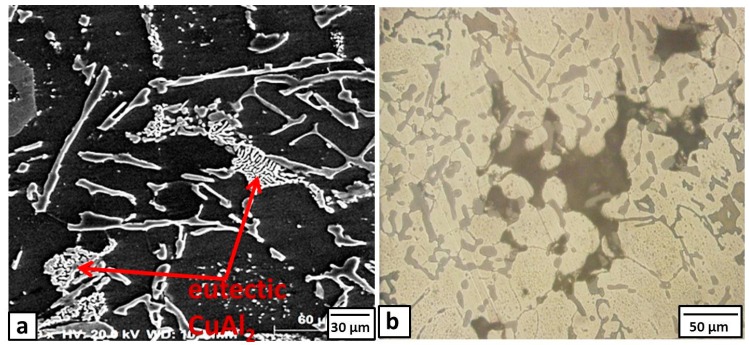
Microstructure of the formed composite after the addition of 10% and 15% Cu; (**a**) SEM Image (10% Cu) and (**b**) optical micrograph (15% Cu).

When increasing the copper powder to 10%, the particles tend to cluster in relatively large patches within the Al grains, as shown in Figure 5a. Most of these Cu particles reacted with the Al matrix, as shown in SEM images in Figure 5a, forming typical eutectic CuAl_2_ intermetallics (shown by arrows in Figure 5a). When the added powder was increased to 15%, many bulk Cu areas or cavities were detected within the matrix as shown in Figure 5b. Based on the present results, we might conclude that there is an upper limit for adding copper powder to the used alloy.

**Figure 6:**
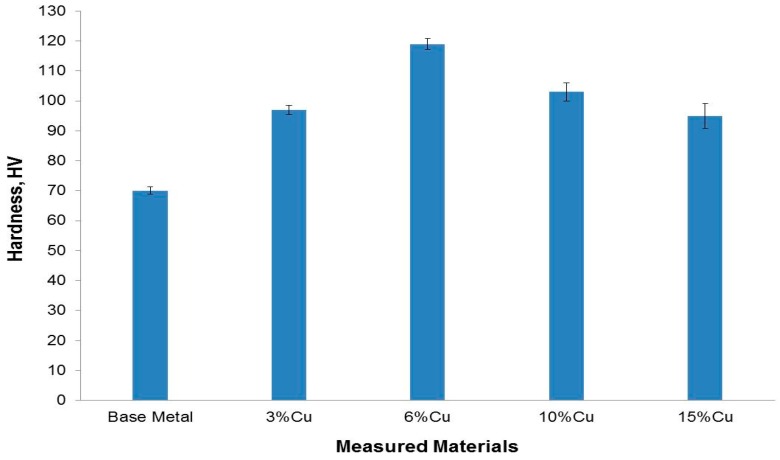
Average hardness measurement values of the Al-12% Si base metal and the formed composites after the addition of copper powder with different weight percentages.

The hardnesses of the Al-Si base metal and the different samples are illustrated in Figure 6. The hardness of the Al-Si base metal (73 HV) is remarkably improved with the addition of copper powder. As the copper addition is increased up to 6%, the hardness value is increased too. The hardness value of the 6% Cu sample is almost twice as high (116 HV) as that of the Al-Si base metal. The significant hardness increment is mainly due to the combined effect of the presence of hard silicon particles throughout the matrix and the presences of the formed *in-situ* hard Cu-aluminides intermetallics, which refine the matrix grains and act as a reinforcing agent.

For the Cu addition >6%, the hardness values showed a decreasing trend, but were still higher than the Al-Si base metal. For example, at 10% Cu, the hardness values reached almost 100 HV, and the hardness value was about 91 HV when the copper addition was 15%. This may be explained by the inhomogeneity and clustering of the copper particles in the Al-Si matrix.

### 3.2. Effect of Heat Treatment

**Figure 7:**
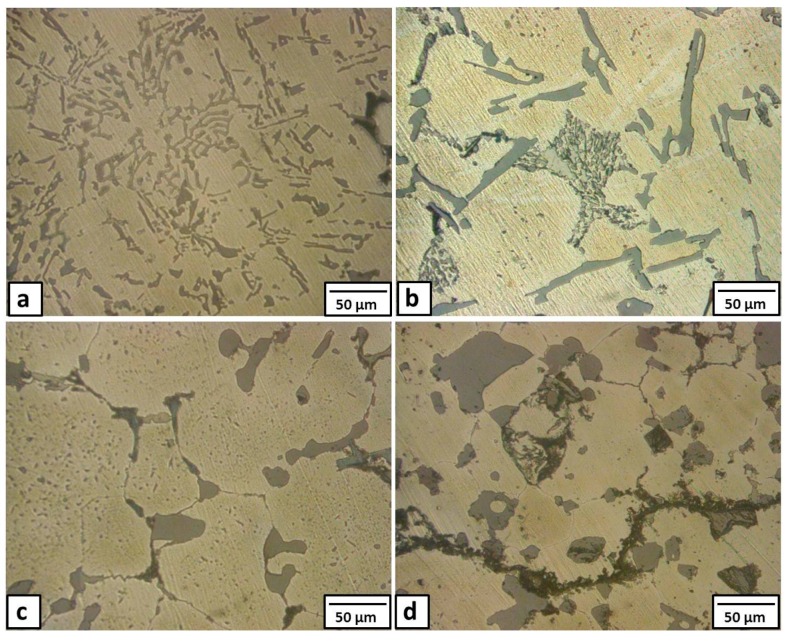
Optical micrographs of the formed composite from the addition of 6% Cu after heat treatment at (**a**) 380 °C for 6 h; (**b**) 380 °C for 18 h; (**c**) 450 °C for 6 h; and (**d**) 450 °C for 18 h.

In this section, the heat treatment was carried out on the formed composite (Sample 2 with 6% Cu added), which showed the highest performance, at temperatures of 380 °C and 450 °C for different holding times (6 and 18 h), followed by air cooling. The microstructural changes occurring during these heat treatments are shown in Figure 7.

**Figure 8:**
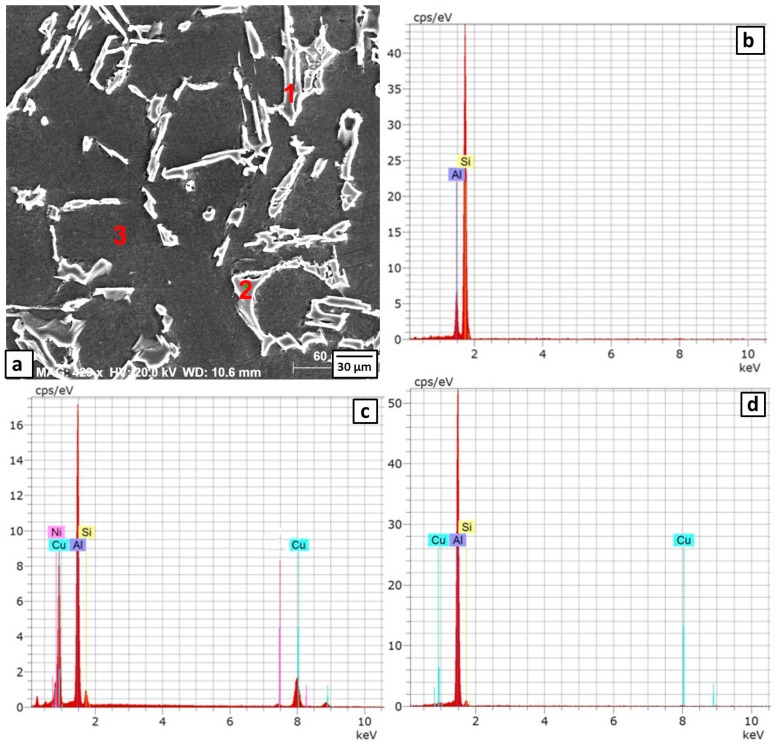
Enlarged SEM image (**a**) of the formed composite of 6% Cu after the heat treatment at 380 °C for 6 h and the EDS spectra of Point 1 (**b**); Point 2 (**c**) and Point 3 (**d**).

**Figure 9:**
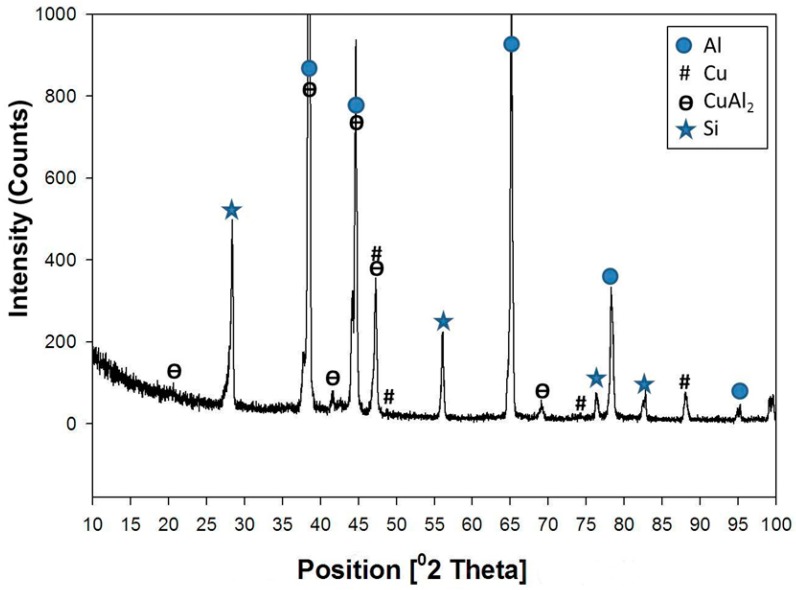
XRD patterns obtained from the formed composite of 6% Cu after the heat treatment at 380 °C for 6 h.

The microstructure of Sample 2 after the heat treatment at 380 °C for 6 h is shown in Figure 7a and Figure 8. The long Al-Si eutectic lamellae are observed at the grain boundaries of fine Al grains (Point 1 in Figure 8a and its EDS spectra in Figure 8b, respectively). Some eutectic CuAl_2_ intermetallics are distributed inside the grains and at the grain boundaries (Point 2 in Figure 8a and its EDS spectra is depicted in Figure 8c). There were no precipitates inside the grains (Point 3 in Figure 8a and its EDS spectra in Figure 8d). These observations were confirmed with XRD patterns as shown in Figure 9. The diffraction peaks of the CuAl_2_ phase showed higher intensities than those observed before heat treatment (compare Figure 9 with Figure 4). The application of the heat treatment at 380 °C for 6 h promoted the reaction of the Cu particles with the Al matrix forming the CuAl_2_ intermetallic.

**Figure 10:**
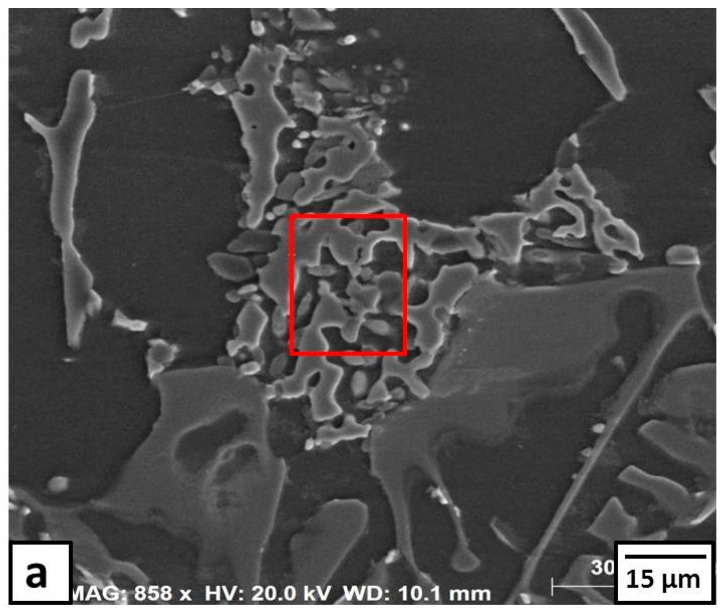

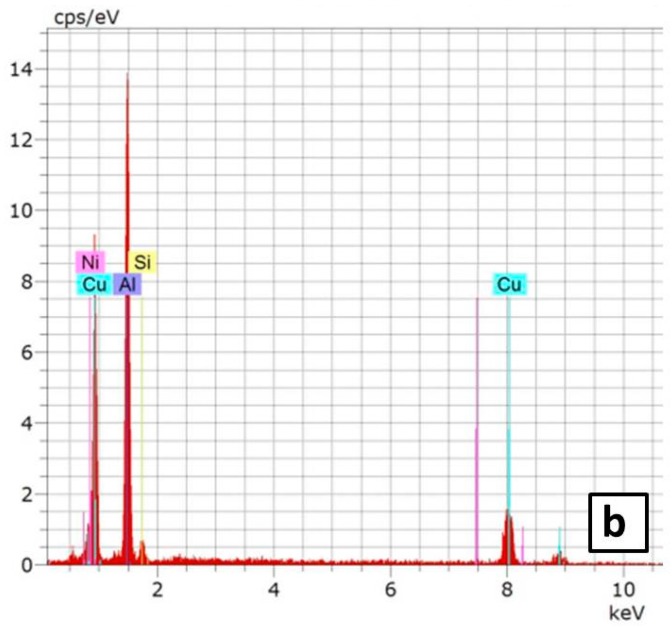
Microstructure and EDS analysis of the formed composite of 6% Cu after the heat treatment at 380 °C for 18 h; (**a**) enlarged SEM image and (**b**) EDS spectra the red area in (**a**).

When the holding time was increased to 18 h at the same temperature (380 °C), the Al grain became larger than before, as shown in Figure 7b. At the same time, typical new eutectic patches were formed as shown in the magnified SEM image in Figure 10a. The atomic concentrations for these patches, as observed from the EDS spectra (Figure 10b), for Cu and Al were 33% and 67%, respectively, thereby suggesting the formation of CuAl_2_ intermetallics.

**Figure 11:**
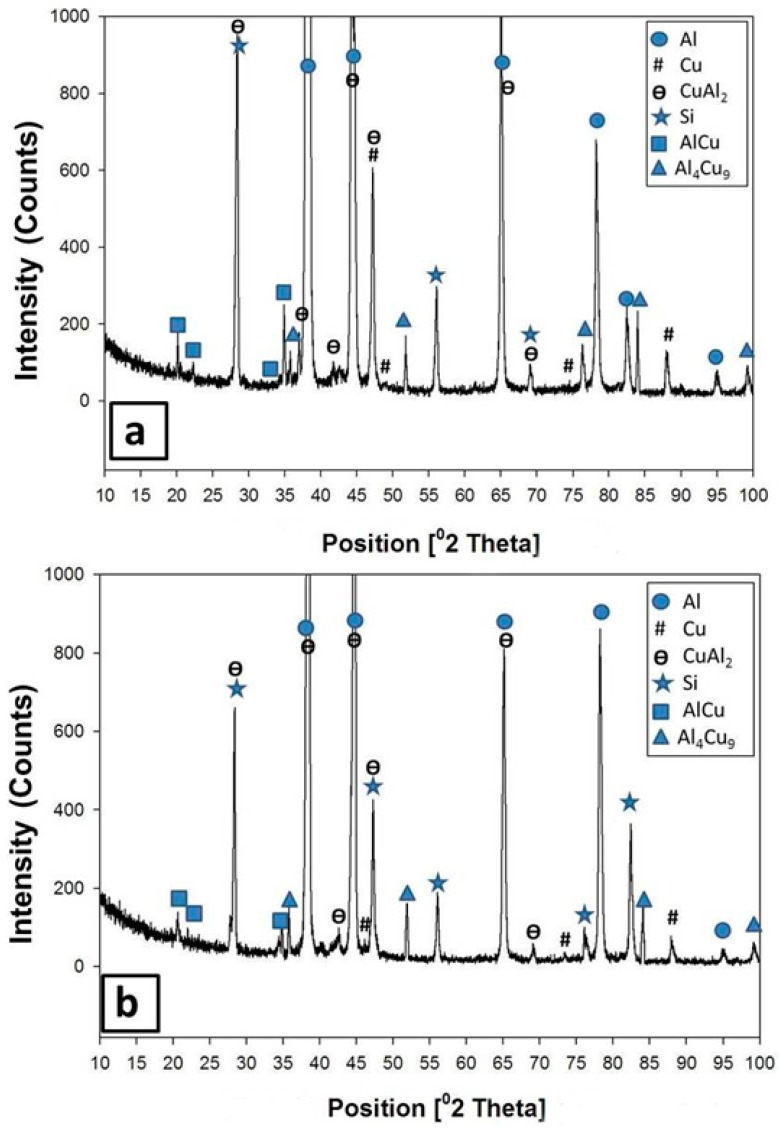
XRD patterns obtained from the formed composite of 6% Cu after the heat treatment at (**a**) 450 °C for 6 h and (**b**) 450 °C for 18 h.

By increasing the temperature of the heat treatment from 380 °C to 450 °C, for 6 h, some drastic changes were noticed both in the microstructure and in the XRD analysis. All the dendritic morphologies almost disappeared in the matrix. Some large particles with curved-like edges were observed at the grain boundaries of large grains, as clearly shown in Figure 7c. Moreover, some small voids were noticed at the triple points of the matrix grains. At the same time, the XRD patterns in Figure 11a showed some peaks of Cu-rich phases (AlCu and Al_4_Cu_9_) beside the CuAl_2_ phase, suggesting that the reaction of the Cu particle with the Al matrix was enhanced with the increase in the heat treatment temperature.

When the holding time was increased to 18 h at 450 °C, most of the eutectics inside the matrix, even the Al-Si, underwent shape perturbations through the heat treatment. The eutectic constituents gradually spheroidized and coarsened with relatively round edges and precipitated at the grain boundaries of large Al grains. Moreover, some cracks were observed in the microstructure as shown in Figure 7d. The XRD analysis after this heat treatment, as shown in Figure 11b, revealed the presence of Al, Si, AlCu, and Al_4_Cu_9_.

**Figure 12:**
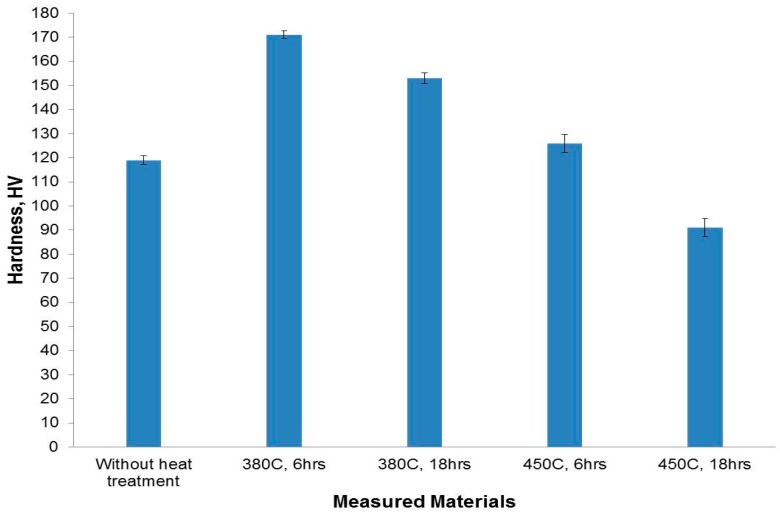
Average hardness measurement values of the formed composites of 6% Cu together with samples heat treated at different temperatures and times.

Figure 12 shows the average hardness measurements obtained at different heat treatment for the 6% Cu addition. Firstly, the hardness values are found to be variable with changing the heat treatment parameters. Heat treatment at 380 °C led to an increase in the hardness to about 170 and 150 HV after annealing for 6 and 18 h, respectively. An increase in the number of fine copper aluminide intermetallic particles that were formed (which dispersed in the fine matrix) led to a strengthening of the material. At the same time, the refining effect of the precipitated particles and the matrix decreased the inter-particle distance, both of which are considered obstacles for the dislocation of movement leading to an increase in hardness. However, the grain coarsening caused by increasing the holding time to 18 h led to decreasing the hardness to a lower value compared to that of the 6 h treatment. 

As the heat treatment temperature was increased to 450 °C, the hardness values was reduced to only 120 HV for 6 h and to less than 90 HV for 18 h. This may be explained by the combined effect of grain coarsening and the existence of some micro-cracks and pores, which resulted in the weakening of the formed structure, especially at a longer holding time.

### 3.3. Wear Resistance of the Developed Materials

**Figure 13:**
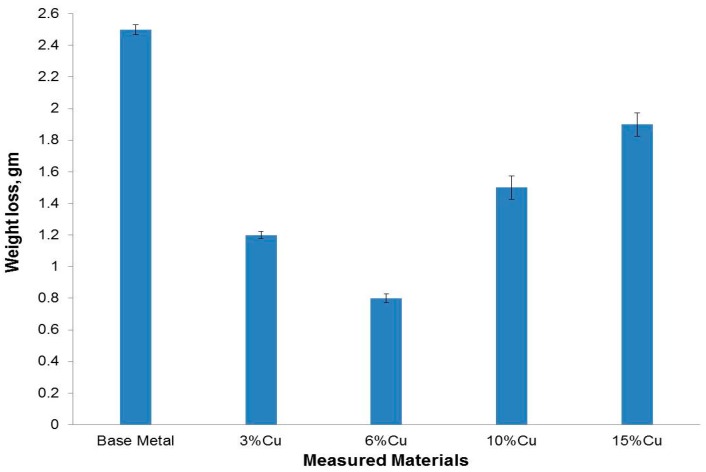
Wear loss values of the Al-12% Si base metal and the formed composites after the addition of copper powder with different weight percentages.

Figure 13 shows the variations of wear weight losses of the developed *in-situ* metal matrix composites together with the Al-Si base metal, after being subjected to a pin-on-disk dry sliding wear test at a fixed load of two bars in air at room temperature. Compared with the Al-Si base metal, all the developed *in-situ* metal matrix composites samples have excellent wear properties under dry-sliding wear test conditions. The weight loss of the 3% added copper sample is almost 50% of the Al-Si base metal sample. This remarkable wear resistance improvement came from the hard, stable, wear resistant fine copper aluminides intermetallic particles, especially CuAl_2_, which homogenously precipitated inside the Al-Si matrix. Moreover, the grain refinement of the matrix also helped in improvement the wear characteristics. 

By increasing the added copper to 6%, the weight loss was decreased to 33% of the Al-Si base metal sample. This may be explained due to the more precipitated fine copper aluminide intermetallic particles within the grain boundaries of the Al-Si matrix. On the other hand, the relatively higher wear rate (lower wear resistance) of the 10% and 15% added copper samples came from the clusters of the added copper particles and due to the cracks that appeared within the microstructure. During the wear test, some patches of these clusters came out which increased the wear rate. At the same time, the cracks permitted and accelerated the scaling of large debris during the wear test.

**Figure 14:**
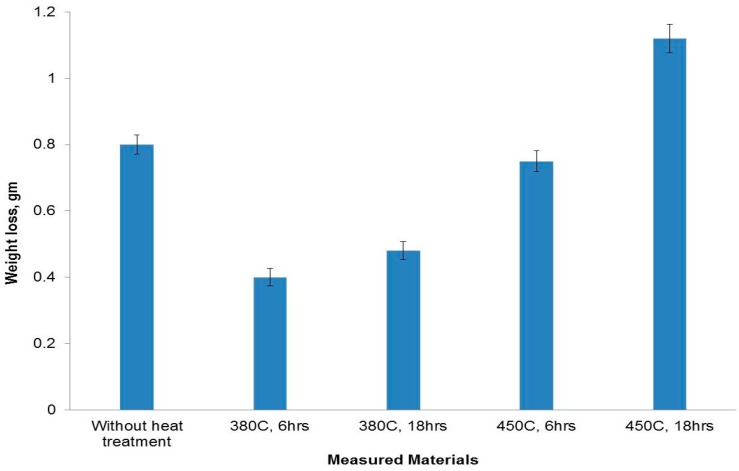
Wear loss values of the formed composites of 6% Cu together with samples heat treated at different temperatures and times.

Regarding the wear behavior of the heat treated 6% added copper, it can be seen from Figure 14 that there was a big difference in wear loss values at different heat treatment temperatures and times. When the temperature and time were 380 °C and 6 h, respectively, the wear weight loss shows a lower value. This is due to the existence of fine hard copper aluminide intermetallic particles aligned in a uniform manner which strengthened the Al-Si matrix and also protected the softer matrix. At the same time, these fine particles showed great effect in refining matrix grains by providing more crystal nucleuses during the solidification phase. The fine particles and matrix resist the cutting into the surface by the counter material. When the holding time was increased to 18 h, the wear loss was slightly shifted to a higher value due to the grain coarsening.

The wear weight loss for samples treated at 450 °C was higher than that of samples treated at lower temperature (380 °C). The increase in the number of hard copper aluminide intermetallic particles formed could not help in improving the wear resistance due to the grain coarsening and the appearance of crack networks within the matrix.

## 4. Conclusions

1. The added copper particles reacted with the Al matrix resulting in the formation of fine copper aluminide intermetallics. Most of the resulted intermetallics were CuAl_2_.
2. The intermetallics that formed were homogenously distributed within the matrix up to the 6% Cu addition.
3. The amount of resulting intermetallics increased after performing heat treatments. At 380 °C, most of the intermetallics were CuAl_2_, while the Cu-rich intermetallics (AlCu and Al_4_Cu_9_) appeared at 450 °C. Increasing the holding time resulted in grain coarsening and formation of some cracks, especially at 450 °C.
4. The hardness of the resulting composite materials was improved. The hardness of the 6% Cu sample without heat treatment was almost twice (116 HV) that of the Al-Si base metal, while the hardness increased up to about 170 HV after heat treating at 380 °C for 8 h.
5. The wear resistance of the resulting composite materials was remarkably improved, especially at lower additions of Cu and at the lower heat treatment temperature.

## Figures and Tables

**Table 1:** The experimental conditions employed in the present study.

Sample No.	Wt % Cu Particles Added to the Matrix.	Holding Temperature, °C	Holding Time, h
1	3	-	-
2	6	-	-
3	10	-	-
4	15	-	-
5	6	380	6
6	6	380	18
7	6	450	6
8	6	450	18
